# The emotional involvement of physicians in the Oncology Intensive Care Unit: a phenomenological-hermeneutic study

**DOI:** 10.3389/fpsyg.2024.1447612

**Published:** 2024-09-06

**Authors:** Debora Tringali, Bernardo Carli, Cosimo Chelazzi, Gianluca Villa, Iacopo Lanini, Antonio Bianchi, Alessandra Amato, Stefano Romagnoli, Rosapia Lauro Grotto

**Affiliations:** ^1^Associazione Lapo APS, Florence, Italy; ^2^Department of Medical and Surgical Specialties, Radiological Sciences and Public Health, University of Brescia, Brescia, Italy; ^3^Department of Health Sciences, Section of Anesthesiology, Intensive Care and Pain Medicine, University of Florence, Florence, Italy; ^4^FILE-Fondazione Italiana di Leniterapia, Florence, Italy; ^5^Department of Health Sciences, Section of Psychology, University of Florence, Florence, Italy

**Keywords:** emotional involvement, intensive care unit, Oncology, physicians, emotional labor

## Abstract

**Background:**

This phenomenological-hermeneutic study is about the experiences of physicians in the Oncology Intensive Care Unit of the Careggi University Hospital, in Florence. The Oncology Intensive Care Unit is a place of great emotional impact and can be create stressful situations. The emotional labor can lead to the development of cynicism, depersonalization and emotional exhaustion. The objective of the study was to learn about and come into contact with the experiences of operators who operate in a highly specialized and critical context.

**Method:**

A semi-structured interview was conducted on 11 physicians in the Oncology Intensive Care Unit of careggi hospital. The interviews were transcribed and subjected to content analysis using the phenomenological-hermeneutic method. The results concerning the emotional involvement of doctors were placed in three macro categories: *difficulties, what helps and needs.*

**Results:**

The interviews highlight the difficulty doctors have in coming into contact with the potentially deadly disease and a further aggravating element appears to be the identification with the patient himself. This condition of difficulty can lead doctors to commit medical errors or to reduce the quality of care.

**Conclusion:**

The results that emerged provide a more detailed understanding of the landscape of emotional reactions of working with the cancer patient in the intensive care unit. In light of the high emotional burden and the inherent possibility of developing burnout in this target population of health care workers, knowing the main critical issues and needs reported may facilitate a more effective tailored intervention.

## Introduction

The Intensive Care Unit (ICU) is among the most advanced units in a hospital, where it is possible to take care of patients with high care complexity. It has the most sophisticated technologies necessary to implement, in a multidisciplinary context, maneuvers to restore or maintain compromised vital functions (De Gaudio and Lanini, 2013).

Practices implemented within a care system of this caliber include haemodynamic monitoring (which refers to the control of blood flow through the cardiovascular system), glycaemic control (as hyperglycaemia damages the immune system, increases endothelial cell apoptosis and causes mitochondrial dysfunction) and oxygenation monitoring (Bersten and Soni, 2010).

There are different types of ICUs: general ICUs and specialized ICUs. In the former, healthcare professionals take care of patients suffering from a wide variety of diseases. The latter, on the other hand, are ICUs that only or predominantly deal with certain diseases. The main types of specialized ICUs are cardiology, neurosurgery, pediatrics, trauma and oncology ICUs. The Oncology Intensive Care Unit represents one of the levels of care and treatment for patients suffering from oncohaematological diseases and presenting organ dysfunction, significant comorbidity or another particularly critical condition. The Oncology Intensive Care Unit is a place of great emotional impact, where people often live a long time before dying, so the professionals working there have to define, on a case-by-case basis, the boundary between the need to intervene in order to preserve survival and the need to avoid futile treatment. It is therefore a complex structure, where medical knowledge, nursing care and instrumental resources meet very high-quality standards and where difficult choices are made (De Gaudio and Lanini, 2013). It is also a place where meaningful relationships are created between the staff, the patients and other family members. The emotional burden of healthcare professionals, who work frantically in a time suspended between life and death, is elevate and the staff must be able to care for the whole person, facing a variety of overt requests and unexpressed needs. The resuscitator’s work in this context is particularly onerous and requires continuous adaptive effort.

The intensive care unit (ICU) can be full of stressful situations for patients, family members, and caregivers. ICU professionals may be emotionally affected by end-of-life issues, ethical decision-making, observing the ongoing suffering of patients, medically unresolving care, lack of communication, and demanding family members of patients. These dynamics can lead to the development of burnout (Embriaco et al., 2007; Van Mol et al., 2015).

Burnout is a term used for a long-term exhaustion with diminished interest usually in the work context, is much more than stress alone, the construct has been defined by Maslach (2009) in 3 dimensions: emotional exhaustion, cynicism and depersonalisation, reduced sense of professional efficacy. Emotional exhaustion refers to the feeling of constant fatigue, the person may feel emotionally drained, without energy and unable to manage their own emotions and those of others. Depersonalisation includes negative and impersonal attitudes and behavior, it may refer to distance and indifference toward colleagues and patients. Finally, low personal achievement refers to decreased work ability, inefficiency and dissatisfaction with work activities (Montgomery et al., 2019; Alvares et al., 2020).

Burnout brings with it numerous repercussions both on a personal level (substance use and abuse, relationship breakdown, sleep disorders, etc.) and on a professional level (consequences on patient satisfaction, reduced quality of care, more frequent medical errors, higher costs for the hospital, etc.) (De Hert, 2020).

The causes of Burnout are multifactorial, generally ascribable to hard work rhythms, salary considered inferior to the workload, great responsibilities, incompatibility with private and work life, feeling of not belonging to the work context (Da Costa and Pinto, 2017; Gonçalves et al., 2019). Several causes can be found as aetiologic factors, distinguished into external (great work pressure, difficulty in collaboration, contradictory instructions, scarcity of resources, absence of social support etc.) and internal (idealistic expectation, perfectionism, always wanting to please other people, suppressing own needs, feeling irreplaceable etc.) (Kleiner and Wallace, 2017; De Hert, 2020).

Working in an intensive care unit, due to the inherent characteristics of the ward, can lead to a greater likelihood of developing burnout than other health professionals, in one study (Guntupalli et al., 2014) it was shown that out of 151 healthcare professionals in an ICU, 54% showed emotional exhaustion, 41% depersonalization and 40% low personal achievement, the results are in line with the literature where the percentage of burnout in ICUs varies from 0 to 70% (De Hert, 2020), working in an intensive care unit was found to be an important prognostic factor for the development of burnout in workers (Alvares et al., 2020). In a review (Chuang et al., 2016) it was shown that in ICU healthcare professionals the Burnout rate varies in a range from 6 to 47%, in the domain of emotional exhaustion the range is between 25 and 61%, in depersonalization between 19 and 45.5% and in low personal achievement between 6 and 59%; whereas being younger than 40 years, being single and childless, having a large workload, night shifts and making end-of-life decisions correlated positively with higher scores on the MBI (Maslach Burnout Inventory).

With regard to the oncology field, a recent review (Yates and Samuel, 2019) pointed out that 32% of oncologists showed emotional exhaustion, 24% depersonalization and 37% low personal achievement, burnout positively correlated with being single, young, poor psychological care, difficulties outside the work context, excessive demands in the work environment as well as a stressful work environment.

A separate consideration concerns decision aids (DAs), tools that support patients in making informed and deliberate decisions about treatment options, which have also been shown to be effective in reducing distress among healthcare professionals. This reduction is mainly due to the decrease in cognitive load and decision conflict that these tools offer. In the oncology context, decision aids play a crucial role in supporting complex therapeutic decisions, considering the multiple factors influencing cancer management, such as tumor characteristics, patient conditions and personal preferences. A study by Green et al. (2019) showed that the use of personalized decision aids for cancer patients improved the quality of clinical decisions by reducing the emotional burden among oncologists through greater clarity and better communication with patients. DAs have been shown to improve the accuracy of risk perception, increase knowledge of possible decisions to be made, change decisions to undergo invasive procedures and elective surgery, and lead to more realistic expectations about the effects of treatment on disease. This is partly due to the increased awareness and better understanding by patients and their proxies of the risks and benefits associated with the decisions to be made (Muehlschlegel et al., 2015).These tools can therefore help to make informed decisions based on the patient’s personal values, thus reducing the emotional burden on healthcare professionals, as well as improving doctor-patient communication (Giles, 2015; Stacey et al., 2017). Despite these promising findings in the literature, however, further research is needed regarding application and standardization in various cultural contexts and in the rigor of studies (Lei et al., 2023).

The results in the literature show that the Burnout syndrome must be recognized and much attention devoted to it due to its numerous implications on clinical practice and quality of life.

From 2017 to 2018, the Chair of Group Psychodynamics of the University of Florence, in collaboration with the Chair of Anesthesia, Resuscitation and Pain Therapy of the University of Florence, promoted a phenomenological-hermeneutic study on the experiences of physicians in the Oncology Intensive Care Unit of the Careggi University Hospital (at Pavilion 16 - San Luca Nuovo), in Florence. The reasons related to the choice of using the phenomenological-hermeneutic method for the analysis of the content that emerged is to be found in the very object of the work: to give space to the participants’ emotions, to encourage them to report on an experience considered unique and placed on a side closer to the world of life and personal experience.

The aim was twofold: on the one hand, to get to know and come into contact with the experiences of operators working within a highly specialized and critical context; on the other hand, to provide them with support, prompting a greater awareness of their resources and the possibilities of mobilizing them (Lauro Grotto et al., 2014).

The results that emerged allow for a clearer picture of clinical work with the oncology patient admitted to the ICU, taking into consideration the subjective experience of the health worker. Attention to both the difficulties experienced and the needs reported can be useful material in delineating what the main critical issues are, representing a reference point for factors that may underlie burnout, thus allowing for a more tailored intervention.

## Materials and method

In the present study, all 11 physicians working in the Oncology Intensive Care Unit of Careggi Hospital decided to take part in the study: six of them were senior doctors, males, aged between 45 and 60, one of whom was the head of the Anesthesiology Department, while five were resident doctors specializing in anesthesia and resuscitation (two in their first year, two in their second year, and one in their third year of specialist training, males, aged between 27 and 32).

Inclusion criteria were: being physicians, working in specialized ICU in contact with cancer patients, having work experience of at least 1 year, knowledge of Italian language.

Exclusion criteria: non-medical healthcare personnel (e.g., nurse), working in the department from less than 1 year, having no contact with oncology patients, lack of consent. The research team conducted an introductory meeting with the staff of the ICU during the morning briefing session: the research team was introducted to the staff members, the aims of the research was discussed, and the successive phases were co-organized. The agreed research plan consisted of four phases: an exploration phase, the testimony collection phase, the content analysis phase and restitution phase.

### Exploratory phase

In the exploratory phase, supervision meetings were held with the ward’s reference figures. The aim was to understand and share the characteristics of such a complex context from an organizational and operational point of view, but also to have some containers for the psychological and emotional burden resulting from contact with “*this world*.” These encounters were fundamental in that they enabled the researchers to become familiar with an unfamiliar and fascinating, yet complex and burdensome context and in order to create a relationship of trust with the team. Moreover, the information thus acquired was useful both for recruitment and for developing the outline for conducting the interviews;

The themes included in the interview were the following:

- The current job (current role, professional background, motivations that led to this choice, difficulties, what is gained from it).
- First contact with the patients and their family members.
- Duration of the care process.
- Definition and communication of the therapeutic plan (who makes the decisions and how they are communicated).
- Problematic conditions or practices, and experiences and feelings associated with them.
- Decision-making and operational roles.
- Relationship with the patient and their family (conflicts within the families and how to handle them).
- Precautions taken toward the family and other patients in anticipation of the patient’s passing.
- Life experience in the 24 h following the patient’s death.
- Feelings experienced upon the patient’s death. Group or personal elaboration of the experience.
- Resources and problems related to patient management: organizational and emotional elements.
- Relationship with other staff members (sharing).
- Description of a relevant experience.

Finally, all the practical issues, such as scheduling the interviews at the most suitable way for the participants, could be resolved by consensus during this introductory phase.

### Testimony collection phase

Having obtained informed consent and research readiness, the doctors were interviewed individually by the researchers (1 senior researcher and 2 junior researchers), all having received appropriate training in the qualitative methods applied, and in particular in the in-depth interview methodology. This tool allows the interviewee to range over the topics and freely recount life experiences. The interviews were audio-recorded and stored by the research team in encrypted form. The interviewees were able to express - without restriction - their experiences, thoughts and opinions, solely guided by the questions posed by the researchers.

The interviews took place in a confidential and suitable context for listening (the meeting room). No limits were set on the duration of the interviews, respecting the participants’ availability (Lauro Grotto et al., 2014). Most of the interviewees expressed themselves casually and enthusiastically, making it clear that the interview was also useful for themselves.

### Processing of results

The written narratives were read out several times individually by the group of researchers (2 seniors and 2 junior) thus beginning the processing process according to the phenomenologic-hermeneutic content analysis method proposed by Montesperelli (1998). The processing of the results was done through inductive method: at first, the individual interviews were processed; tt a second time, the senior researcher traced the differential and peculiar aspects reported by the individual interviewees and were proposed to the other researchers to identify the relevant elements in all the reports, thus reunting them within conceptual categories. In this way, content related to the same themes was systematized into a series of containers. The categories into which the writings were divided, according to the standards defined by the COREQ (Consolidated Criteria for Reporting Qualitative Research) Check List (Tong et al., 2007), have a phenomenological character in that they emerge through the criterion of evidence (Lauro Grotto et al., 2014). The narrative units that emerged from the accounts organized into a series of phenomenological categories (Mantovani and Spagnolli, 2003; Smith, 2003; Reid et al., 2005) were then structured based on an analysis of the unraveling and meaning-making processes of participants’ experiences.

The divergent views of the research team during the analysis and category construction phase were addressed and resolved consensually in an inclusive approach to the different readings. All the interviews were conducted in Italian and only later, during the writing of the article, translated by a native speaker translator, while no back-up translation was done, as according to the authors there was no ambiguity in the proposed translation.

Here we will focus on the results concerning the emotional involvement of doctors, which were placed in three macro categories: *difficulties, what helps and needs.*

### Restitution phase

The results were then fed back to the Oncology Therapy team during a group seminar that took place in three meetings lasting 2 h each: the phenomenologic categories were analyzed and discussed by the participants. As a further development of the research plan the request was advanced to have a specific exploration of life experience during the course of the anesthesiology and resuscitation training.

## Results

### Difficulties

#### Difficulties in finding a balance between the professional and private spheres

Balancing work and private life is very complicated.

Op.6 “*It’s not always easy to unplug, because sometimes you see certain things you say ‘oh my God’, really unpleasant things, people who have really had it all in life.*”

Within the ward, strong bonds can be created between patients and caregivers. These relationships, which are a valuable resource for caregivers, involve a great deal of emotional involvement. Such involvement is experienced as threatening:

Op.7 *“...I used to think that, the way I am, I would be more impressed by the practical side of certain invasive procedures, but I’ve noticed that the thing that I take home the most, that stays with me the most and has the most impact is the psychological aspect of people, of the relationship that is created, it’s not the practical side at all.”*

The search for a work-life balance becomes even more difficult when caring for terminally ill patients:

Op.1 *“Obviously caring for a terminal patient has an impact, as long as you are here and you are busy you are upset about it but you have to get on with the work and everything, but the moment you leave here, for example I keep thinking about certain things, and that definitely has an impact on my mood in general*.*”*

Op.8 *“End-of-life cases leave me with something negative inside, and even when faced with patients who are difficult to manage, who complain or who are even aggressive, I have to say, “I understand!,” and if I see people who are resigned, I honestly feel even worse, and sometimes I see people lying in bed looking into space, and all these things together obviously have an emotional weight. A lot of times you tell the patient, ‘Be patient, try to bear it!’, but it’s really not easy at all, especially knowing that he’s sick, he’s not going to get better, and I do not know how long it’s going to last*.*”*

One never gets used to the death of a patient and each time a strong pain resurfaces:

Op.4 “*There is a continuous overlap between private life and work life, because always having to deal with this kind of patients, and often with patients at the end of life, tends a little bit to attach to you the feelings, the emotions of a workplace. I was once asked “What do you feel when a patient dies?” and I said “I feel like I’m falling into a black hole,” and that’s exactly what it is.*”

#### Immerse yourself

Healthcare professionals must constantly come into contact with the finiteness of the human being:

Op.11 “*A big difficulty is that you see so much death and so you have constant contact with mortality, with the concept of mortality, which then also brings you back to your own mortality. Then there is the also human frustration of seeing the suffering of family members.*”

It therefore becomes impossible not to think about one’s own death:

Op.5 “*I happen to think “I wonder if when I get older I’ll end up here as a patient too!” because it’s inevitable, there’s a lot of things that affect you in here.*”

Mirroring is even more powerful when witnessing the death of a person who has characteristics in common with the carer. As in the case of this interviewee who cared for one of his colleagues on the ward:

Op.7 *“Lately I’ve been thinking about a patient who was hospitalised here for a long time, a colleague by the way, so personally I’ve been empathising more with this situation and being faced with a terminal illness, seeing the person in pain, who is sick and you do not even know if he’s going to get better, the thought of this person remains inside and you think about it even when you go home*.*“*

Or when a patient is the same age as the health professional:

Op.10 *“I see at least one of my peers a month coming through here in a neoplastic situation, even a very compromised one, or in an End of Life state, and I cannot help but think ‘if it happened to him it can happen to me’ but the next step is ‘it’s not happening to me, all the more reason to try to be well’, so it can also be a good thing to realise that things do not always happen only to others*.*”*

#### Emotional involvement in the end of life: the young oncohaematology patient

End-of-life care of young patients is experienced as a strong injustice:

Op.3 *“It is normal for the child to bury the parent, the reverse is against nature. These cases here are the ones one can take home. Because good or bad when the patient dies with very advanced neoplasia, advanced age, big comorbidities, the patient comes to us after a long history of illness, it fits. But for example, I remember a 30-year-old guy that we had here who we sent back to haematology because he had a hyper-acute phase that we managed in a certain way to bypass, then I know that he died ten days later from leukaemia that he discovered overnight because of a stomach discomfort. So here’s a healthy guy who’s basically fine, has a stomach discomfort and then it turns out he has huge lymph nodes and he’s basically a goner, we try chemo, we try pneumonia but in short we all feel it’s an injustice here.*”

It is really difficult to deal with the death of a young patient after a long journey of suffering:

Op.7 *“Dealing with terminally ill patients honestly affects emotions and fatigue a little bit in my opinion, and many times they are elderly patients who have had something from life for better or worse, but sometimes there are young patients who often come from haematology, on whom a lot of work has been done, who are fond of them, and seeing them die is heartbreaking*.*”*

These are stories that leave deep marks:

Op.10 *“I’ve had some situations that objectively I could not shake off, cases in which the patient was haematological, so very young, because it sounds bad to say it but the slightly older patient is a different management, then the family member can live the situation in all its emotional spheres but for us the impact is a little less strong, when you have a slightly younger patient the alarm goes off immediately, then if the patient does not have a positive course even more so, because the news we have to give to the parents becomes more and more loaded, heavier. I remember several cases of this type where one goes away, finishes his shift, but has not completely freed himself, you are left with this negative feeling.*”

They are pervasive experiences that cannot be relegated to the confines of the hospital:

Op.8 “Getting *used to dealing with certain types of patients at the end of life, especially haematology patients who are very young and have a history of serious illness is quite complicated from all points of view, especially from an emotional point of view, it is very difficult to stop thinking about it once you leave here.*”

Dramatic stories to which the mind cannot help but return:

Op.2 *“You take the sick home, there is nothing to do. For example, there was a patient who died in December when I wasn’t there because I was convalescing, a haematology patient with two small children and a husband who was trying to take his own life every two minutes, then there were times when she seemed to be dying and times when she was better, even the last time I saw her I was convinced she was out of danger, but then she died, and this is a nail I drove into my head*.*”*

#### Difficulties in processing experiences

Once out of the hospital, the doctors try to put a damper on the pain, but pain if unrecognized is like water under the ground, sooner or later it resurfaces:

Op.9 *“Our problem is that then you realise that even outside it’s not that people want to hear about dying patients in intensive care, so it’s a topic that you can bring up if you want, but at a certain point you realise it has no appeal. However, the idea as soon as you leave here is to disconnect completely and not hear a bad word of any kind, and this thing here is objectively not the best. You actually try to push these feelings away rather than process them, and in the long run I do not think that does any good. It’s aggravating.*”

Constantly operating in an emergency context makes it even more difficult to process experiences of loss:

Op.2 *“Often you cannot process the whole situation, I personally feel a sensation like being shot out of all these painful emotions, because maybe there are five, six or seven other patients next door that I have to manage. Maybe the alarm rings at that moment, the nurse says ‘Look that one over there has a problem!’, and I have to go, so there is this impossibility to process our pain, our sorrow for a patient who is dying.*”

### What helps

#### The importance of anesthetist rotation

As was repeatedly emphasized by the interviewees, working in the ICU constitutes a constant challenge, not only technically but also emotionally. It then becomes salvific to be able to alternate work in this ward with work in other contexts:

Op.10 *“I realise that in intensive care, in my opinion and from what I see, dealing with patients who tend to become chronic leads to a risk of losing sight of what is really important,* i.e.*, there is a risk of two excesses: either of getting worked up and saying “Absolutely, I said he’s going to make it, so he has to make it!,” or the opposite, of letting go a little and saying “He′s not going to make it anyway.” Whereas having a turnover and doing occasional shifts on the ward allows you to have a fresher outlook, to have that sometimes right and more objective outlook despite the non-continuity, and that for me is a great resource.”*

#### The improvement of some patients

In the face of great technical and emotional commitment, the achievement of an improvement in the patient’s clinical picture and/or his or her recovery is a fundamental motivational drive:

Op.7 *“What*, *however, gives me the impetus to go on is definitely the improvement, that is, when you see that our activity brings a real benefit to the person who then recovers from a critical condition, it is a great satisfaction. And I have seen many people leave so this improvement is perceptible.”*

Op.8 *“Seeing the results even on critically ill patients that you progressively see them improve is a great satisfaction, you see that the procedures you do and the therapeutic efforts lead to a good result, for example haematological patients or septic patients who sometimes stay in intensive care for quite some time, and to see them progressively improve and then leave here in a completely different condition to the one they entered is a great motivational boost because it means that the effort you make has a return.*”

Op.9 *“We actually do battles in the operating theatre, against illnesses and difficult situations, so when you see the satisfaction of the patient and the relative, these little things are the ones that push you to go on looking for another event like that, which you never know when it might happen because in the midst of so many bad days a good day appears.”*

#### The importance of experience

As one doctor on the ward reported, after so many years of working in this field, one slowly comes to realize that the result of good work does not always coincide with the patient’s recovery, but that sometimes when recovery is not possible, it is appropriate to focus on the patient’s quality of life:

Op.9 *“Unfortunately, our work is also this, it’s not that every day we have a positive result, we work on a borderline area, our patients are always serious and when they come to us they are critical, so we have to deal with borderline situations and unfortunately we can often lose. This is something that you understand little by little over the years, because at the beginning, when you come in, you do not really understand this aspect, you come in thinking you are going to revolutionise the world, but then you realise that many situations unfortunately have their own course and you can do relatively little in terms of out how. Then you realise that maybe the results can be other, such as the management of a patient in terms of quality of life.”*

#### Receiving letters of appreciation for end-of-life care

Some interviewees stressed the importance of knowing that the work they do is recognized by the families, even when the patient has passed away.

Op.8 *“Here I realised that even the experiences which ended badly from the clinical point of view, with a deceased patient, carry with them a trove of family members who send notes and thank-you notes even for a long time. And in the letters they more or less all say that on the whole they remember this period as a positive experience because they realised that more than that could not be done, but the context was such as to make the moment as less traumatic as possible, with a protection and closeness on the part of the operators, and this I find very gratifying*.”

Op.10 *“One thing that is really nice is to receive feedback from relatives of deceased patients, because these gestures of affection and gratitude determine the fact that you go beyond medical treatment!”*

#### The importance of having a psychologist on staff

The presence of a dedicated psychologist for patients and their families is a valuable resource:

Op.9 *“In my opinion, if you want to do it, you have to do it by a professional in a structured way, otherwise it loses its effectiveness a little bit, and this, in my opinion, is an Achilles’ heel that is not insignificant, also because I realise that it is also a bit dangerous, because if you cannot manage the emotions that one has while speaking in an interview, then a word may come out that is not good, you may appear nervous and then the family member may perceive this nervousness and turn it into an inadequate treatment of the patient because the doctor is nervous and therefore certainly does not treat the patient well. In other words, misunderstandings can be created, dynamics that do not exist but then inevitably seem real, seem absolutely real. We have the psychologist, J., who gives us a big hand, so when we have a critical family situation or a young patient, we give J. a ring, and he is always available and supports us by making a pathway with the family member separate from us, where we are not present. We, the operators, however, remain uncovered, I mean, ‘And then who will take care of us? Surely we need an external contact person who can be contacted whenever one wants, and who is available to the operators, that would be a great help. I do not know if there already are, but if there are they are apparently not publicised channels. I’ve been saying this for a long time, that sometimes I feel a bit lonely, objectively speaking.”*

### The needs

#### Need for a specific university education dedicated to caring relationships

Most Italian universities do not provide specific teaching in the field of care relationships in their curricula for future health professionals. Healthcare workers therefore find themselves catapulted into a reality with a strong emotional impact, without any tools:

Op.8 *“Initially, we come here and we are completely unfamiliar with it because we do very little practice in the university course, and immediately facing an environment such as intensive care where you have to deal with critically ill patients and often also with patients to be accompanied at the end of life, requires a pathway. Then maybe it’s also a subjective thing and different from person to person, because there is no way in which these aspects are brought to attention in the training course, it’s not planned.*”

Op.9 *“...we do not come out of medical studies prepared on these aspects, we come out very uninformed or at least much more inclined to the clinical and practical aspect of the work and less to the relational aspect. For example, if at the beginning I set recovery as a goal for a patient and I do not achieve it, the fact that this person has experienced a month, a month that he might not have had, in which he has managed not to feel pain and to be in contact with his family, here the importance of this aspect is not immediately understood, in my opinion. In other words, the inauspicious outcome at the beginning was a little frustrating because it cancelled out a little bit the effort one put into it, so one was always asking oneself “where did we go wrong?” or “what else can we do?” In reality, if one then rewound the tape and re-read it, one could see a lot of moments in which the patient had been cared for, his pain had been controlled, he had had moments of human and less professional contact, more relational contact, especially for long-term patients because slowly one overcomes that barrier that exists at the beginning, which is automatically established in the first days between doctor and patient...”*

#### Need for a structured activity where experiences are shared

Living day after day in close contact with the suffering of patients and their families can be wearisome. For some, the need to find a space where they can share their experiences with colleagues is strong:

Op.1 *“In here I would say there is no way to process experiences, or at least, in the circles I have frequented I have never found a reference or even the sensitivity to create one. Vacuous attempts, but really occasional and very limited so I would say no. In general, I believe that in the UK there have already been working groups within intensive care units for a few decades to improve both the quality of life of those who work there and also the quality of communication, but I’m not sure. If there were weekly meetings, for example, decompression chambers one hour a week on Wednesdays from fifteen to sixteen we meet, with whoever wants, and we talk about the working week and everything that happened, it would be very useful. But I do not know if culturally in Italy we are very prepared to do that.”*

#### Need to play down with colleagues

Other medical practitioners, on the other hand, find benefit in being able to deal with emotionally burdensome issues by downplaying them:

Op.10 *“Regarding the need to process certain situations that are a bit heavier in terms of emotional burden, I prefer to talk about it with colleagues because for strange ‘karmic’ reasons outside my reference figures are all people who tend to be anxious/hypochondriacs. On the other hand, with some at work, human relationships of immediate understanding are created. In practice, codes are created between colleagues, a specific language so that sometimes you do not even need too many words, or you even manage to express things with apparent cynicism, knowing that in reality you use that cynicism to lighten yourself and on the other side there is also the understanding of saying ‘you told me in a cynical way, I’ll answer you in an equally cynical way, I understand that it’s difficult to understand how someone can sometimes be ironic about very serious facts, but between colleagues, we understand each other because we all have this way of interpreting things and irony is often very useful.*”

Op.11 *"One sometimes tries to make jokes, to play it down, and I think it's normal because otherwise it's not easy to stay in contact with this kind of thing all the time, but it's not with malice, even saying "that patient is a bit of a pain in the ass", doesn't have a negative meaning because we are all aware that even if the patient complains all the time, that's OK, so these are attitudes that serve a bit to process, and also to buffer the climate that can be created*.*"*

#### Need to share one’s experiences with others

Some interviewees need to be able to tell what they experience inside the ward, to those outside the ward:

Op.4 *"I tend to really need to share both the good and the bad, and in very critical cases I realise that sometimes I really need to say "this happened today, this thing here has triggered me", and I realise that sharing has a positive impact on my mood, my mood improves because it's as if I can lighten the emotional load of certain experiences a little. Then I keep thinking about it, but talking about it with someone who maybe says 'I understand you, it affects me too', it makes you feel less alone in a feeling. I also need feedback to understand how someone else deals with such a strong emotion.*"

Op.7: *"I need to process things, to metabolise them, then of course after a few days this feeling subsides, but I sometimes need to talk about it with someone outside of here, often in the family, it's the only way not to keep it all inside, of course in anonymity and all, but I tell them how I lived through these harrowing experiences for me."*

#### Need for overlap between private and working life

A doctor feels the need to integrate the experiences of his professional life with the experiences of his private life:

Op.4 *“There is no such thing as a pass. I have as many pants and socks in my work locker as I do at home, I shower as much here at work as I do at home, I work as much here as I take work home, I travel with my lunch bag where I have as much food as I have in my fridge at home. There is not a continuity but almost an overlap. And having a continuous overlap between your private and working life makes you appreciate what’s outside much more, in the sense that you often stop for a moment to reflect and say thank you and say “thank goodness I’m able to get out of this place today and go away!,” because always having to deal with this type of patient tends a little bit to attach to you the feelings, the emotions of a workplace. So to be able to maintain contact but experience it in an alternative way through what’s out there is a ray of sunshine in your day. But there’s no story, you must have realised it yourself, there are people here who hate this place but cannot do without it, I mean they just cannot get away from it.”*

#### Need to devote oneself to the vital aspects of existence

Op.4 *“I*’*m very open with people, and I really feel the need to talk to people, I spend my Sundays when I’m free talking to perfect strangers, that is, all the people passing in the middle of the street are fine with me, really! Then I always want to be outside and not in artificial light and artificial air, if it’s cold it’s fine, if it’s hot it’s fine, the important thing is that it’s not a constant temperature and constant light, I really want to get away from that. And then I go in search of what makes me feel good so food basically, lots of food and fresh air! It’s something that has to bring balance back into our lives, because in here you completely lose your spatial references. So getting out of here reminds you that it’s spring outside, reminds you that it’s daytime, reminds you that you are here and now!*”

## Discussion

In the literature, it is known that working in hospital and care settings positively correlates with the burnout syndrome and, in general, with the onset of stress and emotional difficulties (Embriaco et al., 2007; Paiva et al., 2018); this evidence is most pronounced in the oncology setting and especially in the intensive care setting, where Chuang et al. (2016) estimate a burnout rate ranging from 6 to 47% in healthcare workers working in these areas.

In the present study, the results obtained emphasize, through the testimonies collected, the main aspects related to working with cancer patients in intensive care, highlighting the operators’ experiences of the resulting *difficulties, help factors and needs*, which represent the three main macro-categories that emerged through the processing of the interviews (Montesperelli, 1998; Lauro Grotto et al., 2014).

The results show how working with cancer patients in intensive care brings the physician into constant contact with both the physical and psychological suffering of the patient, which inevitably leads to an emotional involvement that is sometimes difficult to confine to the work environment. The difficulty of managing emotional involvement in working with the cancer patient has been reported by Copur (2019) as one of the predisposing factors to practitioner burnout, sometimes leading to complications in the private sphere. In the present study, an experience related to practitioners’ *difficulties* was the confrontation with the topic of illness. Indeed, working constantly and on a daily basis with life-threatening illnesses, sometimes already terminal, leads the practitioner to continually confront the theme of death and suffering. Sometimes the patients’ medical conditions contemplate little margin for hope in recovery, leaving them powerless in the face of the inevitability of the disease’s progression. The constant confrontation with similar situations and the inability to intervene effectively represents a stress factor, which may correlate with the onset of anxiety and depression in the healthcare worker (Eelen et al., 2014), where the worsening of the disease and the death of the patient represent a major emotional criticality (Teixeira et al., 2013; Copur, 2019). In the present study, another factor that emerged as a difficulty was identification with the patient, a phenomenon that may be favored by the presence of characteristics in common with the professional, such as the same age or performing the same job. The emotional involvement of professionals was even more difficult to manage when the patient was young, for example in haemato-oncology, confronting the doctor with a condition of injustice and powerlessness; this critical issue has emerged in other studies in the literature (Nolan et al., 2020).

The analysis of the results revealed several subcategories of protective factors (*helping factors*), one of which is the possibility of shift rotation. Reduced workload, especially at night, is negatively correlated in the literature with the onset of burnout (Yates and Samuel, 2019; Montesperelli, 2001; Ramírez-Elvira et al., 2021). This result is in line with the literature that highlights that flexible work shifts are protective for the development of burnout (Maglalang et al., 2021). The improvement of the patient’s clinical picture represents another protective factor, in fact it allows one to observe in practice the feedback of the efforts made, not to perceive oneself as helpless in the face of the unchanging condition of the hospitalised person, where a perception of passivity and helplessness, correlates with a higher perceived stress in the caregiver (Eelen et al., 2014; Caruso et al., 2012).

Another protective factor that emerged in the results was the importance of work experience, in fact, the ability to understand the nuances in the relationship with in-patients, as well as the real possibilities of intervention, seems to develop with experience in the field and is an element that helps to set achievable goals. This finding is in line with the literature, which shows that there is a negative correlation between burn out and seniority (Chuang et al., 2016; Yates and Samuel, 2019). Contact with patients’ relatives and recognition for the work performed by both patients and relatives has also been reported as a positive factor, which seems to correlate with higher job satisfaction and to be protective in the development of Burnout (Caruso et al., 2012).

Finally, having the possibility of psychological support within the staff, thus a space to confront and contain workload anxieties, was a *helpful factor*, correlating positively with the perceived well-being of the health worker; this finding is in line with the literature on the subject (Yates and Samuel, 2019).

With regard to the *needs that* emerged from the analysis of the interviews, the need to implement appropriate care relationship and communication training in one’s studies was emphasized, a need that is often neglected in favor of specialized medical training; the need for care relationship training has also been reported by other studies in the literature (Girgis et al., 2009; Howlett et al., 2019); communication difficulties with patients and family members can in fact hinder achievable care goals (Piggott et al., 2019).

One need highlighted in numerous interviews was the need to find a space in which to share one’s work experiences with other colleagues, thus the possibility of defusing to channel and manage the emotional reactions elicited by contact with hospitalized patients. Indeed, in the literature, the lack of perceived support between colleagues and a climate of sharing and support correlates positively with the onset of burnout (Maslach et al., 2001; Wåhlin et al., 2010; Yates and Samuel, 2019; Haruna et al., 2022).

Finally, from the analysis of the interviews it emerges how the intensive care context represents for the operators an all-encompassing sphere, both from the point of view of the hours of work performed, and from the psychological point of view, being rich in sensations and emotions that can hardly be relegated to the work environment alone. Some interviews showed how the intensive care environment, precisely because of the pressing work and emotional load, represents a different space–time dimension, a bubble that for its peculiarities often isolates the person from the external environment.

Therefore, one need that emerged was the possibility to devote oneself to the vital aspects of the experience. Indeed, the positive re-evaluation of personal life, taking advantage of the possibility to enjoy its positive aspects, as well as the possibility to reconcile private and working life, represents an essential and protective need for the practitioner (Shanafelt et al., 2015; Ramírez-Elvira et al., 2021).

## Conclusion

The present phenomenological-hermeneutic study explored the emotional experiences of physicians working in the Oncology Intensive Care Unit of the University Hospital of Careggi. The interviews revealed how constant contact with the physical and psychological suffering of patients, often in terminal conditions, leads to significant emotional involvement that is difficult to manage.

It is essential to develop psychological support programs and continuing education to help physicians better manage the emotional burden and prevent burnout. In addition, promoting a healthier work-life balance can help in improving the overall well-being of physicians while ensuring optimal quality of care for patients.

### Clinical implication

The results of the present study extend and deepen the analysis of life experiences of a group of physicians in an Oncology Intensive Care Unit. The issues and difficulties that emerged indicate the need for implementation of dedicated psychological services and staff sharing moments. We believe that attention to the issues that emerged and compliance with them within the institutional setting may be a protective factor to prevent the development of the clinicians’ burnout, therefore providing an indirect support to good clinical practice.

### Study limitations

An initial limitation of the present study may be considered to have interviewed only physicians, not including other healthcare personnel, which may nevertheless have brought out additional clinical implications and nuances useful for understanding the general working climate. A further limitation is having considered the subjective experience of physicians in working with cancer patient, without having explored the effect of using artificial intelligence and decision aids in clinical practice (Triberti et al., 2021): despite the fact that these tools certainly influence the emotional factors examined in working with the patient and family members, none of the participant spontaneously mentioned this issue during the interview. It is hoped that further studies will fill this gap.

## Data Availability

The raw data supporting the conclusions of this article will be made available by the authors, without undue reservation.
